# Ultrasound-Guided Needle Aspiration vs. Surgical Incision and Drainage of Breast Abscesses: Our Experience at a Tertiary Care Centre in North India

**DOI:** 10.7759/cureus.103189

**Published:** 2026-02-08

**Authors:** Omar M Bhat, Fazl Ul Haq, Iqra Yousuf

**Affiliations:** 1 General and Minimal invasive Surgery, Sher-i-Kashmir Institute of Medical Sciences (SKIMS), Srinagar, IND; 2 SKIMS Soura, Sher-i-Kashmir Institute of Medical Sciences (SKIMS), Srinagar, IND; 3 Anesthesia, Sub District Hospital Seer, Srinagar, IND

**Keywords:** breast abscess, fistula, recurrance, surgical incision drainage, usg guided

## Abstract

Background

For women, breast abscesses are a common morbidity and an emergency. It typically affects women who are breast feeding/nursing. The traditional approach to treating a breast abscess involved making a surgical incision and draining the abscess. This procedure was closely linked to the requirement for general anaesthesia, a lengthy recovery period, frequent dressing changes, challenges with breastfeeding, and potentially disappointing cosmetic results. Ultrasonography is a valuable diagnostic tool that helps to identify breast abscesses, directs the positioning of the needle during aspiration, and allows for the detection of many abscess loculations, all of which are helpful when aspirating breast abscesses with a needle.

Methods

This prospective comparative study was conducted in the Department of General and Minimal Invasive Surgery, Sher-i-Kashmir Institute of Medical Sciences (SKIMS), to compare surgical incision and drainage with ultrasound (US)-guided needle aspiration in patients with breast abscesses during a two-year period. All the involved patients were screened, and a detailed history and physical examination was collected. Diagnosis of breast abscess was made after confirming by ultrasonography of both breasts. The study participants were randomized into two groups after having their full written informed consent for both types of interventions.

Results

Out of 118 patients, 60 (50.8%) underwent surgical incision and drainage, and 58 (49.2%) underwent ultrasound-guided needle aspiration. Most patients were in the reproductive age group (30-40 years), and lactating women constituted a substantial proportion in both groups. Recurrence was observed more frequently in the ultrasound-guided aspiration group (12.0%) compared to the incision and drainage group (5.0%); however, most recurrences in the aspiration group were successfully managed with repeat aspirations. The incision and drainage group had a significantly higher mean duration of illness (10.36 ± 2.50 vs. 8.06 ± 2.83 days; p=0.012) and a higher incidence of fistulization and significant scarring. Cosmetic outcomes were significantly better in the ultrasound-guided aspiration group, with lower rates of disfigurement (p<0.05)

Conclusion

It was concluded that USG-guided aspiration of breast abscess is a better and more cost-effective intervention as compared to surgical incision and drainage as a treatment option in the management of breast abscesses for both puerperal and non-puerperal breast abscesses.

## Introduction

Breast abscess is a common morbidity and an emergency condition among women, most frequently affecting lactating mothers. Mastitis occurs in nursing women with an incidence ranging from approximately 3% to 20% [1]. It is defined as a painful, erythematous, warm, and inflamed area of the breast, accompanied by flu-like symptoms and fever exceeding 38.5°C [2].

The incidence of breast abscesses in the Indian subcontinent ranges from 0.4% to 11%. These abscesses are commonly associated with the gestational and lactational period, whereas non-lactational breast abscesses are relatively uncommon in India.

Management of breast abscess continues to pose a significant clinical challenge. In the early stages of mastitis, antibiotic therapy alone may be sufficient. With advances in imaging techniques, ultrasound-guided percutaneous needle aspiration has emerged as a minimally invasive alternative for the management of breast abscesses [3].

Traditionally, breast abscesses were managed by surgical incision and drainage, which had long been considered the standard method of treatment [4]. However, this approach is associated with the need for general anesthesia, prolonged healing time, frequent dressing changes, difficulty in continuing breastfeeding, and potentially unsatisfactory cosmetic outcomes.

Further refinement of minimally invasive techniques, including ultrasound-guided aspiration combined with cavity irrigation and instillation of local antibiotics along with systemic antibiotic therapy, has demonstrated improved success rates in selected patients [5]. Several studies have reported favorable outcomes with ultrasound-guided needle aspiration compared to surgical incision and drainage, citing reduced morbidity, shorter hospital stay, faster recovery, and better cosmetic results [6,7].

However, larger abscesses - particularly those with a mean diameter of ≥3 cm or a volume of approximately ≥21.5 mL - have been shown to have lower success rates when treated with aspiration and irrigation alone, without local antibiotic instillation.

The research question addressed by this study was whether ultrasound-guided needle aspiration provides superior clinical and cosmetic outcomes compared to surgical incision and drainage in patients with breast abscesses.

## Materials and methods

This prospective comparative study was carried out for a two-year period from July 2022 by the Department of General and Minimal Invasive Surgery, Sher-i-Kashmir Institute of Medical Sciences (SKIMS), with ethical permission obtained by the institution vide approval No. SIMS 131/ IEC-SKIMS/2022-449. After obtaining informed consent, a total of 118 patients with a diagnosis of breast abscess who presented to the general and minimally invasive surgery department were included in the study. 

All patients aged 12 years and above with ultrasonography (USG) documented breast abscess who were willing to participate in the study were included, as the institution routinely manages adolescent breast pathologies; no pre-pubertal children were included in the study. Patients with tubercular abscess, chronic granulomatous mastitis, galactocele, malignancy, or fungal infection were excluded after confirmation by culture sensitivity and/or histopathological examination, where indicated.

A thorough medical history was gathered, and all the patients involved underwent evaluation. After a thorough history, a clinical examination, and ultrasounds of both breasts, the diagnosis was confirmed.

**Figure 1:**
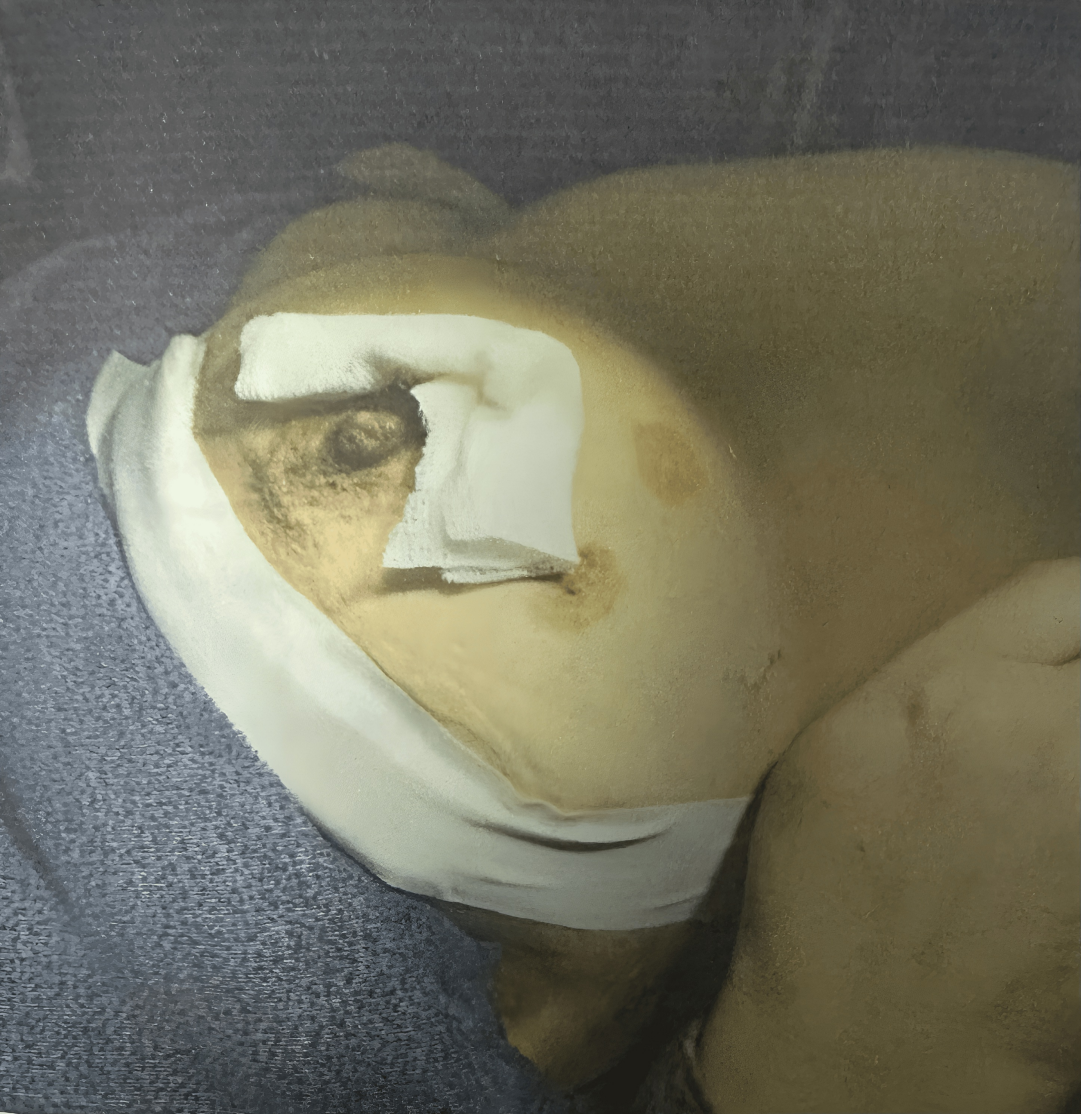
Postoperative picture after surgical incision and drainage of a breast abscess

**Figure 2:**
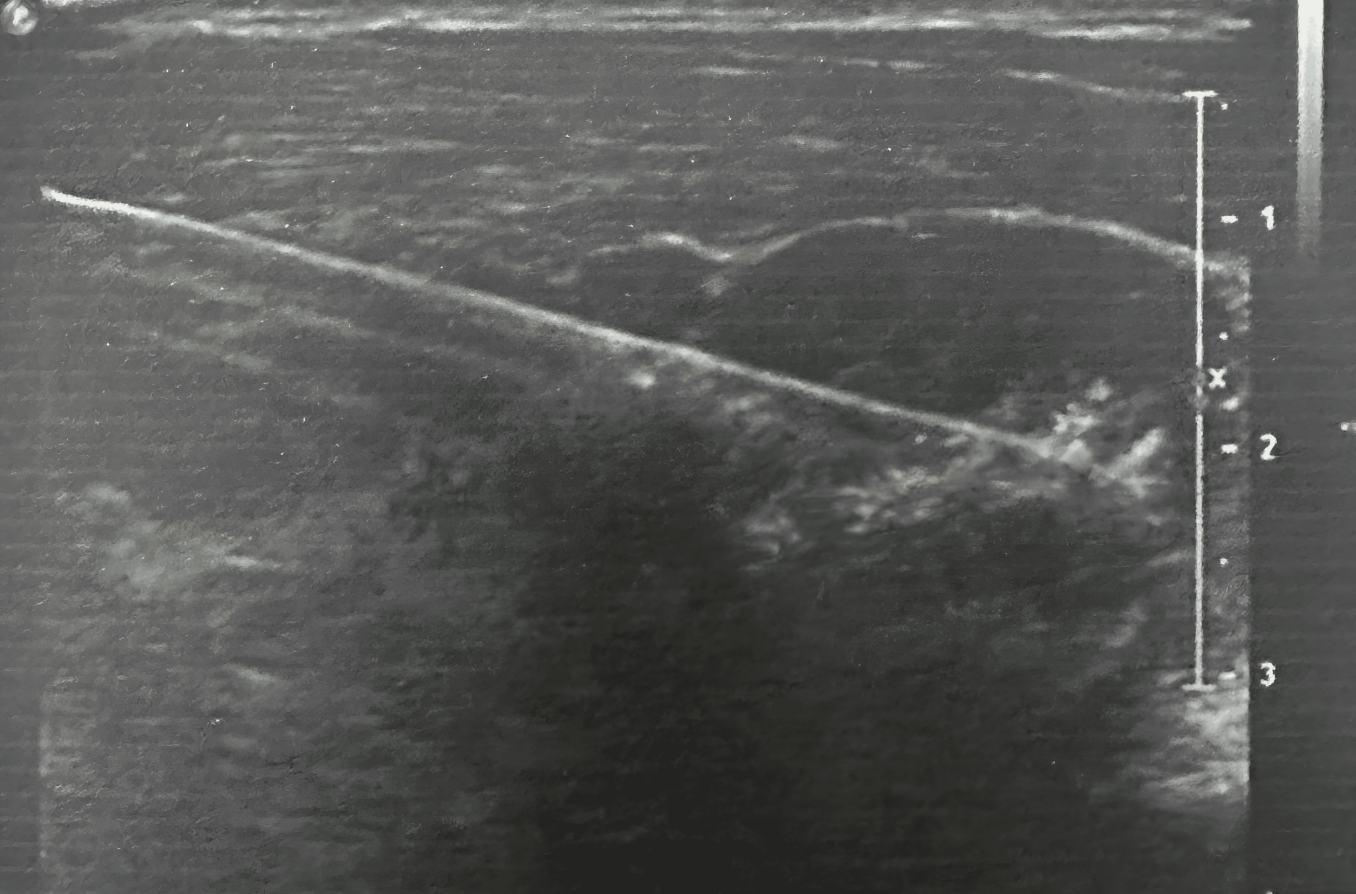
Ultrasound-guided drainage of a breast abscess

After confirmation of breast abscess by ultrasonography and obtaining informed consent, patients were randomly allocated into two groups using a simple random allocation method. Group 1 underwent surgical incision and drainage, while group 2 underwent ultrasound-guided needle aspiration. Due to the nature of the interventions, blinding of the treating surgeon was not feasible; however, allocation was performed without prior knowledge of outcomes. Group 1 underwent an incision and drainage (Figure 1), while group 2 had the abscess cavity aspirated or re-aspirated under USG guidance (Figure 2).

The administration of suitable antibiotic coverage was provided to all patients, mostly through injections of 500 mg of metronidazole intravenously thrice a day and 1 g of amoxicillin twice a day (ATD). Patients from both groups had their pus cultures and sensitivity tests performed, and the medications were then adjusted accordingly. On post-procedure days three and seven, an ultrasound examination was performed to rule out any residual abscess.

In order to determine which treatment modality for breast abscess was superior, both groups were compared using outcome parameters commonly reported in previous studies [6,7], including residual abscess, duration of recovery, recurrence, procedure-related complications, cosmetic outcome, and return to breastfeeding in lactating mothers. To evaluate clinical improvement, every patient in the study was re-evaluated two weeks after being discharged. The data was collected using an Excel spreadsheet (Microsoft, Redmond, Washington), and statistical analysis was performed using SPSS v 29 (IBM Inc., Armonk, New York). Continuous variables were expressed as mean plus-minus standard deviation and compared using an independent t-test. Categorical variables were expressed as frequencies and percentages and compared using the Chi-squared test. A p-value of <0.05 was considered statistically significant.

## Results

Out of the 118 patients in our study, 60 (50.8%) underwent surgical incision and drainage, and 58 (49.2%) had USG-guided needle aspiration. The youngest patient included in the study was 22 years old, while the oldest patient was 55 years old. In our study, most of the participants were in the reproductive age with 63 (53.3%) of patients in the age group of 30-40 years followed by 30 (25.4%) in the age group of 20-30 years, 22( 18.6%) in the age group of 41-50 years and three (2.5%) older than 50 years. Among the incision and drainage (I&D) group, 23 (44%) subjects were lactating, and among the ultrasound-guided needle aspiration group, 30 (46%) subjects were lactating. There was a slightly higher proportion of abscesses on the left side, with 59 (58.4%) of patients having left-sided breast abscesses.

**Table 1:** Depicting comparison of two study groups

Parameters		Group 1: incision and drainage	Group 2: USG-guided aspiration
Age	20-30	12 (10.2%)	18 (15.2%)
	31-40	34 (28.8%)	29 (24.6%)
	41-50	12 (10.2%)	10 (8.5%)
	>50	2 (1.7%)	1 (0.8%)
Lactational status	Lactating	23 (19.5%)	28 (23.7%)
	Non-lactating	37 (31.4%)	30 (25.4%)
Site	Right side abscess	26 (22%)	21 (17.8%)
	Left side abscess	34 (28.8%)	25 (21.2%)

In our study, the USG-guided aspiration group had a greater rate of recurrence (7; 12%) in comparison to the group that underwent surgical incision and drainage (3; 5%). Higher chances of antibioma formation, which was noted in three (5.1%) patients undergoing aspiration and not seen in the incision drainage group. Participants in the surgical incision and drainage group had a mean sickness duration of 12.06±2.83 days, as compared to participants in the USG group, which was significantly higher, with a mean duration of 10.36±2.5 days of illness. Patients managed with ultrasound-guided needle aspiration were predominantly treated on an outpatient basis, whereas patients undergoing incision and drainage required inpatient admission for postoperative care and wound management and were mostly discharged on postoperative day one. Additionally, the I&D group had a significantly higher proportion of severe scarring than the USG group. A higher proportion of minimal scarring was seen in the USG group, and there was also a significantly higher proportion of fistulisation in the I&D group (5; 8.3%) compared to no fistulisation in the USG group, as depicted in Table 2. The type of intervention was found to have a statistically significant correlation with the length of the illness, scarring, disfigurement, and fistulisation (p<0.05).

**Table 2:** Long-term outcome of two study groups

Parameter	Group 1: incision and drainage, n (%)	Group 2: USG-guided aspiration, n (%)	Statistical test used	Test value	p-value
Significant scarring	9 (15.0)	2 (3.4)	Chi-square test	χ² = 9.6	0.002*
Fistulisation	5 (8.3)	0 (0)	Fisher's exact test	-	0.024*
Recurrence	3 (5.0)	7 (12.0)	Chi-squared test	χ² = 1.9	0.168
Chronic pain	2 (3.3)	2 (3.4)	Fisher's exact test	-	0.972
Disfigurement	7 (11.6)	1 (1.7)	Fisher's exact test	-	0.031*
Minimal loss of breast tissue	3 (5.0)	0 (0)	Fisher's exact test	-	0.084
Significant loss of breast tissue	2 (3.3)	0 (0)	Fisher's exact test	-	0.160
Antibioma formation	0 (0)	3 (5.1)	Fisher's exact test	-	0.074
Mean duration of illness (days), mean ± SD	10.36 ± 2.50	8.06 ± 2.83	Independent t-test	t = 4.7	0.012*

## Discussion

Breast abscess is a common clinical condition, predominantly affecting women of reproductive age, particularly during the lactational period. Traditionally, surgical incision and drainage (I&D) has been considered the standard treatment modality. However, with advances in imaging and minimally invasive techniques, ultrasound-guided needle aspiration has emerged as an effective alternative, aiming to reduce morbidity, hospital stay, and cosmetic deformity [3,4].

In the present study, the majority of patients belonged to the reproductive age group, with most cases occurring between 30 and 40 years of age, followed by the 20-30-year age group. These findings are consistent with previous studies that have demonstrated a higher incidence of breast abscesses among women in their childbearing years, particularly lactating mothers [8].

The mean duration of illness prior to intervention was significantly higher in the incision and drainage group compared to the USG-guided aspiration group, suggesting delayed presentation or advanced disease severity in surgically managed patients [8]. Recurrence was observed in both groups; however, patients in the USG-guided aspiration group were successfully managed with repeat aspirations, avoiding surgical intervention in most cases [9,10].

Previous studies have demonstrated that ultrasound-guided treatment of breast abscesses in lactating women is effective and associated with high patient satisfaction and preservation of breastfeeding [11-13].

Cosmetic outcome was significantly better in the USG-guided aspiration group. A statistically significant association was observed between scarring and type of intervention, with higher disfigurement rates among patients undergoing incision and drainage [12]. Although complete resolution was achieved in most patients undergoing USG-guided aspiration, a subset required multiple aspirations, especially in large or multiloculated abscesses. Antibioma formation was observed in a small proportion of patients, consistent with a previous report [13]. 

Milk fistula formation, a distressing complication particularly in lactating women, was observed exclusively in patients undergoing surgical drainage, while no such complication occurred in the USG-guided aspiration group [14].

Overall, the findings of this study reinforce existing evidence that ultrasound-guided needle aspiration is an effective, cosmetically superior, and patient-friendly alternative to surgical incision and drainage in appropriately selected patients [11-14].

Limitations

The present study was conducted at a single tertiary care center, which may limit the generalizability of the findings. Additionally, long-term follow-up was limited. Larger multicentric studies with extended follow-up are recommended to further validate these results.

## Conclusions

The current prospective study came to the conclusion that a variety of characteristics, including procedure ease, practicality, acceptance, duration for full healing, and cosmetic and functional outcome, should be taken into consideration when choosing a treatment strategy. Participants in the I&D group had a considerably longer mean duration of illness than those in the USG group, and the I&D group had a significantly higher proportion of severe scarring than the USG group. USG-guided aspiration of breast abscesses is a better and more cost-effective intervention as compared to surgical incision and drainage as a treatment option in the management of Breast Abscess for both puerperal and non-puerperal breast abscesses.
